# Saliva as a diagnostic frontier: Assessing healthcare students’ readiness for salivary biomarkers in early cancer detection

**DOI:** 10.1371/journal.pone.0332717

**Published:** 2025-09-30

**Authors:** Rajalakshimi Vasudevan, Taha Alqahtani, Saud Alqahtani, Afaf Aldahish, Praveen Devanandan, Geetha Kandasamy, Tasneem Asiry, Samar Ahmed Alqarni, Futoon Musaed Alsultan, Taif Nasser Mohammed Alharrani, Fay Abdulrahman, Fatimah Hassan

**Affiliations:** 1 Department of Pharmacology, College of Pharmacy, King Khalid University, Abha, Aseer, Kingdom of Saudi Arabia; 2 Department of Pharmacy Practice, St. Peter’s Institute of Pharmaceutical Sciences, Hanamkonda, Telangana, India; 3 Department of Clinical Pharmacy, College of Pharmacy, King Khalid University, Abha, Kingdom of Saudi Arabia; University of Pennsylvania, UNITED STATES OF AMERICA

## Abstract

**Background:**

Salivaomics represents a novel diagnostic domain that provides a non-invasive and accessible alternative to conventional cancer detection techniques. Saliva encompasses a diverse array of indicators, such as microRNAs, cytokines, and proteins, indicative of both systemic and oral health. Notwithstanding the increasing data endorsing its clinical efficacy, salivary diagnostics are still underutilised, mostly owing to insufficient knowledge among prospective healthcare practitioners.

**Objective:**

This study aimed to assess the knowledge, attitude, and perception (KAP) of healthcare students in Saudi Arabia regarding the use of salivary biomarkers for early cancer detection, and to identify factors influencing their willingness to integrate such diagnostics into the curriculum.

**Methods:**

A cross-sectional study was conducted using a structured, self-administered questionnaire distributed among 221 students from medicine, pharmacy, dentistry, and nursing programs. Data were analyzed using descriptive statistics, chi-square tests, t-tests/ANOVA, Pearson correlation, and logistic regression.

**Results:**

Overall, 68.8% of students had heard of salivary biomarkers, though only 42.1% could correctly identify specific biomarkers. Attitudes toward saliva-based diagnostics were overwhelmingly positive, with mean scores above 4.0 on key items such as test feasibility and willingness to recommend their use. Key barriers included lack of curricular integration (68.78%) and concerns about test accuracy (53.39%). Significant associations were found between knowledge levels and willingness to incorporate the topic into academic programs (p = 0.017). Logistic regression identified predictors of willingness, including higher knowledge scores, female gender, older age, internship status, and enrollment in medicine or pharmacy.

**Conclusion:**

Healthcare students showed a positive attitude toward saliva-based cancer diagnostics but demonstrated notable knowledge gaps, particularly in biomarker identification and diagnostic validity. These findings support integrating salivaomics into healthcare curricula, alongside faculty development and targeted training, to improve readiness for clinical adoption.”

## Introduction

Cancer continues to be a significant concern in global health, with both incidence and mortality rates consistently increasing. The GLOBOCAN 2020 report estimated 19.3 million new cancer cases and nearly 10 million cancer-related deaths globally (Sung et al., 2021), with recent trends further underscored in *Cancer Statistics 2023* (Siegel et al., 2023).” These concerning statistics underscore the pressing necessity for enhanced early detection methods to alleviate the worldwide cancer burden. Timely diagnosis not only enhances survival rates and treatment efficacy but also markedly decreases healthcare expenditures and improves patients’ quality of life [1–4].

Historically, cancer diagnosis has predominantly depended on imaging techniques, histopathological examination, and serum biomarker testing [5]. Although these approaches are considered the gold standard, they frequently provide challenges due to their intrusive nature, high costs, and logistical difficulties, especially concerning repeated testing and widespread screening. Furthermore, these strategies may establish obstacles to patient compliance, particularly in marginalised populations and resource-limited environments [6]. As a result, researchers have been investigating more accessible, non-invasive diagnostic alternatives, with saliva emerging as a highly promising medium for biomarker-based cancer diagnosis.

Saliva is a readily obtainable fluid that necessitates no invasive techniques. It contains numerous clinically relevant constituents, including DNA, RNA, microRNAs (miRNAs), proteins, metabolites, cytokines, and extracellular vesicles. These components indicate the health of both the oral cavity and the body, rendering saliva valuable for diagnosing and monitoring various ailments, including cardiovascular issues, immunological disorders, and malignancies. The utilisation of sophisticated technologies such as mass spectrometry, next-generation sequencing, and microfluidics has enhanced the precision and sensitivity of biomarker detection in saliva [7,8].

Recent literature has shown that salivary biomarkers can identify several cancer types with notable diagnostic precision. Elevated levels of microRNAs such as miR-21 and miR-125a have been detected in the saliva of patients with oral squamous cell carcinoma (OSCC) and breast cancer, suggesting their potential utility for non-invasive diagnostics [9–12]. Additionally, salivary levels of lactate dehydrogenase (LDH), tumor suppressor protein p53 antibodies, interleukins (IL-6 and IL-8), and various exosomal proteins have been associated with tumor activity in cancers of the lung, pancreas, and gastrointestinal tract [13,14].

Saliva-based diagnostics present numerous benefits compared to conventional blood-based techniques. The process of collection is straightforward, carries minimal risk of infection, and does not necessitate specialised personnel, rendering it suitable for high-throughput screening and for use among paediatric, geriatric, or needle-averse populations. Additionally, saliva collection is generally less resource-intensive than blood sampling and can be conducted in remote or under-resourced environments. However, in certain cases, transportation and storage may still require a cold chain to preserve protein stability and prevent degradation [15]. Even with these advantages, the broad implementation of salivary diagnostics in clinical settings remains constrained, in part due to insufficient awareness and confidence among healthcare professionals and students.

Recent literature has emphasized the importance of integrating innovative diagnostic technologies into healthcare education to ensure readiness for clinical adoption. Zolotov et al. (2022) [16] and Li et al. (2021) [17] highlight how inclusion of novel diagnostics in medical and pharmacy curricula enhances competency, fosters acceptance, and accelerates clinical translation. These principles equally apply to salivary biomarker diagnostics, underscoring the need for curricular integration. Also, recent reviews emphasize the readiness and curricular integration needs around saliva-based diagnostics [18–20], underscoring salivaomics as a practical platform for training and clinical translation. The effective adoption of new diagnostic technologies relies not just on their scientific validation but also on the comprehension and acceptance by the upcoming healthcare professionals. Regrettably, current studies indicate a notable deficiency in understanding salivary biomarkers among students in the healthcare field. A study conducted by Aro et al. [18] highlighted that a limited number of dental and medical students were aware of the significance of salivary diagnostics in cancer detection, even though they showed a desire to gain further knowledge on the topic. A further investigation conducted by Kaczor-Urbanowicz et al. (2019) [20] emphasised the absence of salivaomics integration within medical and dental education as an obstacle to its clinical application.

Students in the healthcare field embody the future of medicine, dentistry, pharmacy, and nursing—individuals who will take on the crucial roles of applying diagnostic technologies and informing patients. The comprehension, mindset, and viewpoint regarding non-invasive diagnostic tools such as salivary biomarkers play a vital role in influencing future clinical practices and public health strategies. Enhancing understanding within educational settings could lead to quicker adoption, better diagnostic methods, and greater acceptance among both patients and healthcare providers.

This study seeks to evaluate the knowledge, attitude, and perception (KAP) of healthcare students regarding salivary biomarkers for the early detection of cancer, considering the relevant factors involved. The objectives are:

I. Assess students’ foundational understanding of the characteristics and diagnostic value of salivary biomarkers. Assess their perspectives on the trustworthiness and practicality of saliva-based tests in relation to conventional blood-based diagnostics.
II. Recognise the obstacles to adoption, including insufficient awareness, doubts about scientific validity, or limited availability in clinical environments.

This investigation holds significant importance within the framework of Saudi Arabia, where academic establishments are actively working to incorporate cutting-edge health technologies into their educational programs. Identifying the gaps and challenges allows the study to offer actionable recommendations for enhancing educational programs and training that facilitate non-invasive diagnostics. “This study aims to evaluate the role of salivary biomarkers in cancer detection and monitoring, with the objective of providing evidence to support their integration into precision medicine and routine healthcare practice.”

## Methodology

### Study design

This study employs a cross-sectional, descriptive design utilising a structured questionnaire. The design effectively facilitates the evaluation of knowledge, attitudes, and perceptions (KAP), enabling comparative analysis among various academic groups.

### Study population

Participants include undergraduate and postgraduate students enrolled in healthcare-related programs (medicine, dentistry, pharmacy, and nursing) at accredited academic institutions. We select these disciplines based on their involvement in cancer diagnosis, patient management, and public health education.

### Inclusion criteria

a. They must be currently enrolled in an academic programme related to healthcare.
b. The participant must be at least 18 years old.
c. Ability to understand and complete the survey in English.
d. Participants must voluntarily agree to participate and provide informed consent.

### Exclusion criteria

a. Students from non-healthcare disciplines, such as business and engineering, are not eligible.
b. Licensed healthcare professionals who are already in practice are also excluded.
c. Those with prior training or advanced knowledge in salivary diagnostics, such as lab researchers involved in salivary biomarker studies, are also excluded..
d. Incomplete or partially filled questionnaires.

### Sampling technique and sample size

A **convenience sampling** strategy were employed. A minimum sample size of 221 participants was determined based on a power analysis (α = 0.05, power = 0.80, medium effect size d = 0.5). This ensured sufficient statistical power and representation across different academic disciplines. “Recruitment was carried out through university notice boards, online student forums, emails, and class announcements

### Data collection instrument

The **tool is a set questionnaire that participants fill out themselves, organised into five parts, based on the study’s goals and earlier tested** KAP models in healthcare research [21]:

a. **Demographics**: Collects data on age, gender, academic discipline, and year of study.
b. **Knowledge Assessment:** Consists of close-ended questions about salivary biomarkers, their applications in cancer detection, and comparisons with blood-based tests. Knowledge scores were calculated by assigning 1 point for each correct response and 0 points for incorrect or ‘don’t know’ responses. The total knowledge score was then categorised as follows: low awareness (<50% of maximum score), moderate awareness (50–74%), and high awareness (≥75%).
c. **Attitude and Perception**: It assesses students’ acceptance, confidence, and trust in saliva-based cancer diagnostics using a 5-point Likert scale.
d. **Barriers and Challenges**: Identifies perceived obstacles, such as lack of awareness, doubts about test accuracy, or preference for conventional methods.
e. **Open-**ended questions: allow participants to provide additional concerns or recommendations regarding the inclusion of salivary diagnostics in healthcare practice and education.

The questionnaire is expected to take **5–10 minutes** to complete and will be distributed digitally via Google Forms or printed hard copies depending on accessibility.

### Data access period

Data for this study were accessed over a defined period from 15 April 2025–25 May 2025 for research purposes. During this time, responses submitted via the online questionnaire platform were systematically collected, anonymized, and organized for statistical analysis. This access period ensured adequate response rates and data integrity, in accordance with ethical approval requirements and institutional guidelines.

### Pilot testing and validation

A pilot test involving 10–15 students was conducted to assess clarity, flow, and internal consistency. Feedback obtained was used to refine the questionnaire. Cronbach’s alpha values were calculated and added. The reliability was strong (α = 0.81 for attitude, α = 0.84 for perception). Content validity was ensured through expert review by faculty in dentistry, clinical pharmacology, and public health.

### Data analysis

Quantitative data were entered into statistical software such as SPSS v25.0 for analysis. Descriptive statistics (mean, standard deviation, frequency, and percentage) were used to summarise demographic data and KAP scores. Inferential statistics included **Chi-square tests** to assess associations between categorical variables (e.g., knowledge level and field of study). Independent samples t-tests or ANOVA were applied to compare the mean scores among different groups while the Pearson’s correlation was used to investigate the relationships between knowledge, attitudes, and perception scores. A p-value of **<0.05** was considered statistically significant.

### Ethical considerations

The research was adhere to ethical guidelines outlined by the Declaration of Helsinki. Ethical approval was obtained from the Research Ethics Committee (HAPO-06-B-001) at King Khalid University under approval number ECM#2025−703 (09 April 2025).”. Informed consent were obtained electronically or in writing before participation. The survey does not collect any personally identifiable information, and all responses were kept strictly confidential.

## Results

A total of **221 healthcare students** participated in the study. The demographic distribution of participants, including gender, age, and field of study, is summarized in **Table 1**. The majority were aged between **20–25 years (59.73%)**, followed by those over 25 years (27.60%) and 18–20 years (12.67%). In terms of gender, **females constituted the majority (68.78%)**, while **males made up 26.24%**. Pharmacy students represented the largest academic group (40.27%), followed by medicine (23.98%), nursing (19.46%), and dentistry (16.29%). Most respondents were either interns (30.32%) or in their fourth year (24.89%).

**Table 1:** Demographic profile of participants (n = 221).

Variable	Category	Frequency (n = 221)	Percentage (%)
**Age**	18–20	28	12.67%
	20–25	132	59.73%
	>25	61	27.60%
**Gender**	Male	58	26.24%
	Female	152	68.78%
	Other/Prefer not to say	11	4.98%
**Field of Study**	Medicine	53	23.98%
	Dentistry	36	16.29%
	Pharmacy	89	40.27%
	Nursing	43	19.46%
**Year of Study**	First	32	14.48%
	Second	26	11.76%
	Third	41	18.55%
	Fourth	55	24.89%
	Intern	67	30.32%

### Knowledge of salivary biomarkers (Table 2)

There were variations in knowledge scores among students in different fields, as presented in **Table 2**. The majority of students (68.8%) had heard of salivary biomarkers for cancer detection. When asked about the applicability of these biomarkers for specific cancers like oral, breast, or lung cancer, **56.6% answered correctly,** while **28.1% answered incorrectly** and **15.3% were unsure**. Only **42.1% could correctly identify known salivary biomarkers** such as miRNA, LDH, and CRP, indicating knowledge gaps. Additionally, **47.5% of students** correctly recognized that salivary diagnostics could be as accurate as blood-based diagnostics.

**Table 2:** Knowledge assessment summary.

Knowledge Question	Correct (%)	Incorrect (%)	Not Sure (%)
**Have you heard of salivary biomarkers for cancer detection?**	152 (68.8%)	56 (25.3%)	13 (5.9%)
**Can salivary biomarkers detect oral/breast/lung cancer?**	125 (56.6%)	62 (28.1%)	34 (15.3%)
**Recognized salivary biomarkers (miRNA, LDH, CRP)?**	93(42.1%)	84(38.0%)	44 (19.9%)
**Accuracy of salivary vs. blood-based diagnostics**	105 (47.5%)	81 (36.7%)	35 (15.8%)

### Attitudes and perceptions

Students demonstrated generally positive attitudes, with item-level details provided in **Table 3**. Most participants agreed that **saliva-based tests are a good alternative to blood tests (mean = 4.2, SD = 0.81)** and would recommend their use for early cancer detection (mean = 4.1). Trust in the scientific validity of salivary biomarkers scored a mean of **3.9,** and **non-invasive testing was seen as a tool to boost screening uptake (mean = 4.4).**

**Table 3:** Attitudes toward Saliva-based diagnostics.

Attitudinal Statement	Mean	SD
**I believe saliva-based tests are a good alternative to blood tests.**	4.2	0.81
**I would recommend saliva-based tests for early cancer detection.**	4.1	0.89
**I trust the scientific validity of salivary biomarkers.**	3.9	1.05
**Non-invasive saliva tests could increase cancer screening rates in hospitals and clinics.**	4.4	0.76

### Perceived barriers

The leading barriers to adopting salivary diagnostics are summarized in **Table 4**, led by insufficient curricular awareness (68.78%) and concerns about diagnostic accuracy (53.39%). **limited clinical availability (43.89%)**, and **preference for traditional methods (35.75%)**. Peer or faculty skepticism was reported by **29.41%.**

**Table 4:** Perceived barriers to adoption.

Barrier	Frequency (n)	Percentage (%)
**Lack of awareness in curriculum**	152	68.78%
**Concerns about test accuracy**	118	53.39%
**Limited clinical availability**	97	43.89%
**Faculty/peer skepticism**	65	29.41%
**Preference for traditional methods**	79	35.75%

### Willingness to integrate into curriculum

A significant **79.64% of students expressed willingness** to include salivary diagnostics in healthcare curricula as shown in Table 5. Only 8.60% were opposed, and 11.76% remained unsure.

**Table 5:** Willingness to include in curriculum.

Response	Frequency (n)	Percentage (%)
**Yes**	176	79.64%
**No**	19	8.60%
**Not Sure**	26	11.76%

### Demographic influences on attitudes

Statistically significant differences in attitude scores were observed based on gender (p = 0.038), age (p = 0.021), year of study (p = 0.045), and field of study (p = 0.032). Interns, those over 25, and students in medicine and pharmacy had the most favorable attitudes as mentioned in Table 6.

**Table 6:** Mean attitude score by demographic groups.

Demographic Variable	Group	Mean Attitude Score	SD	p-value
**Gender**	Male	3.91	0.85	0.038
	Female	4.22	0.78	
**Age Group**	18–20	3.82	0.90	0.021
	20–25	4.16	0.82	
	>25	4.33	0.76	
**Year of Study**	First	3.74	0.91	0.045
	Second	4.02	0.80	
	Third	4.15	0.75	
	Fourth	4.36	0.70	
	Intern	4.47	0.68	
**Field of Study**	Pharmacy	4.18	0.83	0.032
	Medicine	4.39	0.75	
	Dentistry	4.01	0.79	
	Nursing	3.89	0.88	

* Independent t-tests for Gender and ANOVA for Age and Year and field of Study.

### Knowledge vs. willingness

Chi-square test to assess association between knowledge and willingness as shown in the Table 7. It revealed a significant association between knowledge level and willingness to incorporate salivary diagnostics into the curriculum (p = 0.017). Students with high knowledge scores were more likely to support curricular integration.

**Table 7:** Cross-tabulation of knowledge level vs. willingness to include in curriculum.

Knowledge Level	Yes (n)	No (n)	Not Sure (n)	Total (n)	p-value
**Low (0–1 correct)**	40	10	18	68	0.017
**Moderate (2–3)**	82	5	4	91	
**High (4 correct)**	54	4	4	62	

### Predictors of willingness – logistic regression

As mentioned in the Table 8, binary logistic regression showed that knowledge score (OR = 1.45; p = 0.005), being female (OR = 1.82; p = 0.043), older age (OR = 1.65; p = 0.037), being an intern (OR = 2.10; p = 0.019), and enrollment in pharmacy (OR = 1.78; p = 0.041) or medicine (OR = 2.05; p = 0.014) significantly predicted willingness to include salivary biomarkers in academic curricula.

**Table 8:** Binary logistic regression – predictors of willingness.

Predictor Variable	Odds Ratio (OR)	95% CI	p-value
**Knowledge Score**	1.45	1.10–1.91	0.005
**Female (vs. Male)**	1.82	1.01–3.28	0.043
**Age > 25 (vs. 20–25)**	1.65	1.02–2.98	0.037
**Intern (vs. First Yr)**	2.10	1.12–3.94	0.019
**Pharmacy (vs. Nursing)**	1.78	1.02–3.10	0.041
**Medicine (vs. Nursing)**	2.05	1.15–3.66	0.014
**Dentistry (vs. Nursing)**	1.63	0.92–2.88	0.087

## Discussion

The present study provides new insights into healthcare students’ perceptions of saliva-based diagnostics for cancer detection in Saudi Arabia. Our findings demonstrate a moderate level of awareness but generally favorable attitudes, reflecting both the promise and the challenges of integrating this emerging diagnostic modality into future clinical practice. This discrepancy between enthusiasm and actual knowledge underscores the importance of aligning educational strategies with students’ receptivity toward novel technologies.

Our cohort’s patterns of moderate awareness and favorable attitudes are consistent with previous studies, including Aro et al. (2020) [18] and Kaczor-Urbanowicz et al. (2019) [20], which also reported high levels of acceptability but variable depth of factual knowledge among trainees. By extending these observations, our study identifies demographic predictors—knowledge score, female gender, and clinical seniority—associated with greater willingness to adopt salivary diagnostics. These findings reinforce the view that positive attitudes alone are insufficient unless supported by adequate training and curricular reinforcement [7,22].

Importantly, our results revealed that while students were receptive to salivary diagnostics, knowledge gaps persisted, particularly in identifying specific biomarkers and understanding diagnostic accuracy. Similar gaps have been observed among European dental students (Aro et al., 2020) [18]. Addressing these deficiencies requires structured, targeted educational interventions. Evidence from Vasudevan et al. [19] on oral health literacy among Saudi university students demonstrates that structured learning initiatives can significantly enhance both comprehension and acceptance of emerging healthcare practices. Adopting similar curricular approaches for saliva-based diagnostics could bridge the current deficits and ensure students are adequately prepared for clinical application.

Consistent with earlier studies [4–6,13], our participants strongly acknowledged the non-invasive, accessible, and patient-friendly nature of saliva as a diagnostic medium, with preference over blood-based testing. The high mean attitude scores parallel the findings of Cortes-Reynosa et al. [23], who noted that younger healthcare professionals particularly value innovations that reduce patient burden and enhance compliance.

Regression analysis further highlighted that knowledge level, gender, academic seniority, and field of study significantly predicted willingness to adopt salivary diagnostics. Female students and those in clinical years, especially interns, demonstrated higher acceptance. These trends mirror previously reported gender-based differences in healthcare technology acceptance, suggesting that social and experiential factors may shape receptivity toward new diagnostic tools [21].

At the same time, our study identified key barriers. The most prominent was insufficient curricular exposure (68.78%), followed by concerns about diagnostic accuracy (53.39%). Notably, less than half (42.1%) of participants could correctly identify specific salivary biomarkers [7,23]. These findings strongly point to a curricular gap, where content on biomarker science and diagnostic validity is either absent or insufficiently emphasized. This aligns with Kaczor-Urbanowicz et al. [20], who argued that the absence of salivaomics within formal medical education significantly limits clinical adoption. Embedding such content into curricula, alongside practical demonstrations, could accelerate acceptance and application.

In summary, our findings suggest that while healthcare students in Saudi Arabia express clear enthusiasm toward saliva-based diagnostics, knowledge deficits and curricular insufficiencies remain substantial barriers. Systematic integration of salivaomics into medical and allied health education could not only enhance awareness and competence but also empower future practitioners to champion these tools in clinical practice. Ultimately, such efforts could contribute to improved early detection rates, patient outcomes, and broader adoption of saliva-based diagnostics in healthcare systems.

### Strengths and limitations

This study presents several strengths. Notably, it is the first of its kind conducted in Saudi Arabia to focus on multidisciplinary healthcare students, offering novel insights into this population. The research employed robust statistical analyses, including logistic regression and cross-tabulations, enhancing the reliability of its findings. Furthermore, the study provides a comprehensive evaluation by addressing both cognitive (knowledge) and affective (attitude) domains, contributing to a well-rounded understanding of the subject.

However, the study also has some limitations. The cross-sectional design restricts the ability to infer causal relationships between variables. Moreover, the use of convenience sampling, while practical for recruiting a diverse student cohort, introduces the possibility of selection bias and limits the representativeness of the findings. Students who chose to participate may differ in motivation or awareness compared to the broader healthcare student population, which should be considered when interpreting the results. The reliance on self-reported data introduces the potential for response bias. Lastly, the findings are based solely on student input without validation from faculty members or practicing clinicians, which may affect the depth of interpretation.

### Future directions and implications

This study highlight several key areas for advancement. Educational institutions are encouraged to integrate modules on salivaomics and non-invasive diagnostics into undergraduate curricula to better prepare future healthcare professionals. Interventional studies should be conducted to assess the impact of educational programs on knowledge, attitudes, and practices (KAP) related to salivary diagnostics. Additionally, cross-cultural comparisons could provide valuable global perspectives on the readiness of various regions to adopt such technologies. Faculty development is equally important, with targeted training programs needed to equip educators with the expertise to effectively teach and mentor students in this emerging field. Future work should also include qualitative research involving faculty members, curriculum designers, and practicing clinicians to complement the student perspectives obtained in the current study. Such interviews and focus group discussions could provide valuable insights into perceived barriers, resource requirements, and strategies for incorporating salivary diagnostics into existing healthcare curricula. This approach would allow for a more comprehensive understanding of institutional readiness and would inform the development of practical, evidence-based implementation plans for educational reform. Finally, findings from this and similar studies can inform national health education policies, supporting initiatives aimed at early cancer detection and the broader implementation of non-invasive diagnostic strategies.

## Conclusion

This study demonstrates that healthcare students show strong interest and moderately good awareness regarding salivary biomarkers for early cancer detection. The findings underscore a **positive attitudinal shift toward non-invasive diagnostics**. However, critical knowledge gaps and curricular deficiencies persist. Bridging these through educational reform and increased clinical exposure is essential to fully harness the promise of saliva-based cancer diagnostics in the next generation of healthcare professionals.

## Supporting information

S1 File Raw dataset underlying the results presented in the manuscript, including [briefly describe contents – e.g., patient demographics, experimental measurements, survey responses, etc.]. (XLSX)
